# Carcinome épidermoïde de la vessie: expérience rétrospective dans un hôpital universitaire marocain et revue de la littérature

**DOI:** 10.11604/pamj.2020.37.143.23540

**Published:** 2020-10-08

**Authors:** Youssef Kadouri, Imad Boualaoui, Salim Lachkar, Hachem El Sayegh, Lounis Benslimane, Yassine Nouini

**Affiliations:** 1 Service d’Urologie A, CHU Ibn Sina, Faculté de Médecine et de Pharmacie, Université Mohammed V Souissi, Rabat, Maroc

**Keywords:** Vessie, tumeur rare, carcinome, épidermoïde, Bladder, rare tumor, carcinoma, squamous cell

## Abstract

Le carcinome épidermoïde de la vessie est une variété rare de tumeurs vésicales qui représente moins de 5% des cancers de vessie. Son sex ratio est équilibré et la population noire semble préférentiellement atteinte. Sa présentation clinique est non spécifique et dominée par l´hématurie. La résection endoscopique de la vessie avec un examen anatomo-pathologique permet de poser le diagnostic. Le traitement du carcinome épidermoïde de la vessie reste sujet à de nombreuses controverses du fait de la rareté des cas rapportés dans la littérature. Cependant le traitement de choix semble être une cystectomie totale avec curage ganglionnaire étendu. Nous rapportons une série de 10 cas de carcinome épidermoïde qui ont été traités et suivis au sein de notre service. Notre analyse est basée sur l´évaluation des caractéristiques épidémiologiques, cliniques, anatomo-pathologiques, et thérapeutiques du carcinome épidermoïde de la vessie, ainsi que sur l´étude des aspects évolutifs et des facteurs pronostiques.

## Abstract (en)

Squamous cell carcinoma of the bladder is a rare form of bladder tumor, accounting for less than 5% of bladder cancers. Sex ratio is balanced and the black population is more affected by this disease. It manifests with non-specific clinical symptoms and it is dominated by haematuria. Diagnosis is based on endoscopic resection of the bladder with anatomopathological examination. Treatment remains controversial because of the rarity of cases reported in the literature. However, radical cystectomy with extensive lymph node dissection is the treatment of choice. We here report a series of 10 cases of squamous cell carcinoma treated and followed up in our department. Our study highlights the epidemiological, clinical, anatomopathological, therapeutic and evolutionary features of squamous cell carcinoma as well as its prognostic factors.

## Introduction

La tumeur de vessie représente le 2^e^ cancer le plus fréquent du tractus uro-génital après le cancer de la prostate [1], son incidence est de plus de 300 000 cas par an dans le monde ce qui représente 5 à 8% de tous les cancers [2]. Le carcinome urothélial transitionnel représente 90-95% des tumeurs malignes de vessie [3]. Les tumeurs non urothéliales de la vessie, aussi bien bénignes que malignes, forment des entités beaucoup plus rares et représentent moins de 5% de tous les néoplasmes vésicaux, parmi lesquelles on trouve le carcinome épidermoïde qui représente le 2^e^ cancer par ordre de fréquence après le carcinome urothéliale [4]. C´est une entité rare, qui demeure méconnue dans les pays occidentaux, contrastant avec une incidence élevée au Moyen-Orient et en Afrique de l´Est. Son sex ratio est équilibré et la population noire semble préférentiellement atteinte.

## Méthodes

Notre travail est une étude rétrospective des dossiers médicaux, étalée sur une période de 10 ans concernant dix cas de carcinome épidermoïde de la vessie, diagnostiqués, traités et suivis au Service d´Urologie A du CHU de Rabat. Notre analyse est basée sur l´évaluation des caractéristiques épidémiologiques, cliniques, anatomo-pathologiques, et thérapeutiques du carcinome épidermoïde de la vessie, ainsi que sur l´étude des aspects évolutifs et des facteurs pronostiques. Les données recueillies pour la réalisation de ce travail proviennent des dossiers des patients dans les archives du service et des comptes rendus opératoires.

## Résultats

Il s´agit de 7 hommes et 3 femmes, l´âge moyen était de 60,4 ans avec des extrêmes allant de 41 ans à 72 ans. Un antécédent de tabagisme chronique a été trouvé chez 5 patients (50%), avec une consommation moyenne de 32 PA. Une irritation vésicale chronique a été retrouvée chez 5 de nos 10 malades soit dans 50% des cas: un patient a été opéré pour un calcul vésical, un patient a bénéficié d´une périnéostomie pour traumatisme de l´urètre, un patient a été suivi pour sténose de l´urètre post-infectieuse pour laquelle il a bénéficié d´une urétrotomie endoscopique 4 fois, une patiente a été suivie pour infection urinaire chronique, et un patient avait une preuve histologique de Bilharziose urinaire. Sur le plan clinique, le maitre symptôme était une hématurie totale avec des caillots, retrouvée chez tous les patients, associé à des signes irritatifs du bas appareil urinaire dans 70% des cas, des signes obstructifs dans 40% des cas (4 malades), une altération de l'état général (AEG) avec anorexie et amaigrissement non chiffré dans 40% des cas (4 malades). L´examen clinique a révélé une masse hypogastrique chez un malade, des fistules avec issue d´urines et de pus chez 2 malades, et une sensibilité hypogastrique chez un autre.

**Figure 1:**
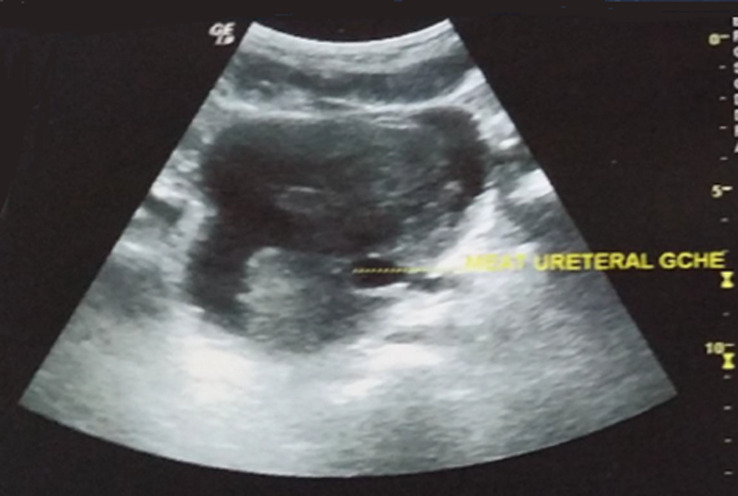
cliché d´échographie montrant un processus tumoral à large base d´implantation trigonal et pariétale gauche, englobant le méat urétéral homolatéral

**Figure 2:**
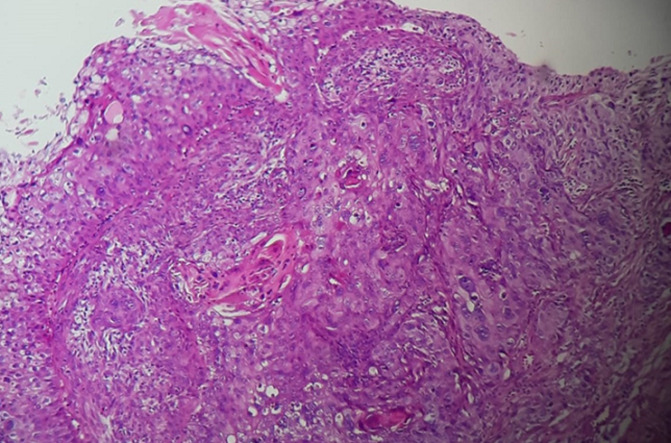
carcinome épidermoïde moyennement différencié et kératinisant de la muqueuse vésicale (HE x200)

**Figure 3:**
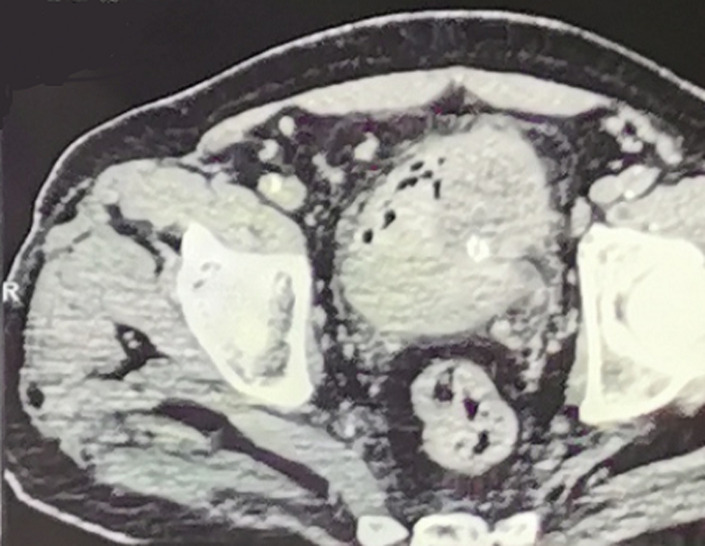
cliché scannographique montrant une énorme tumeur occupant la quasi-totalité de la vessie

Sur le plan paraclinique: une anémie a été retrouvée chez 4 patients, soit 40%, avec un taux d´hémoglobine allant de 4 g/dl à 16 g/dl, secondaire à la maladie néoplasique, ainsi qu´à la spoliation sanguine par hématurie. Cette anémie a imposé une transfusion sanguine dans 4 cas soit chez 40% des patients. Une insuffisance rénale a été retrouvée chez deux patients. Elle s´est amélioré après drainage par néphrostomie percutanée. Sur le plan radiologique: tous nos patients ont été examinés par une échographie réno-vésicale qui avait permis d´explorer la morphologie vésicale et de suspecter le diagnostic d´une tumeur de vessie en objectivant une masse tissulaire pariétale (Figure 1). La résection trans-urétrale de la vessie (RTUV), a été faite chez tous nos patients au service, elle a été complète chez 7 patients et incomplète chez 3 car tumeur jugée incontrôlable endoscopiquement. Elle a été unique chez tous les patients. L´examen anatomo-pathologique des copeaux de résection a permis de confirmer le diagnostic de certitude de carcinome épidermoïde de la vessie chez tous nos malades (Figure 2). Le scanner thoraco-abdomino-pelvien dans le cadre du bilan d´extension a été réalisé chez tous nos patients et a révélé: Une extension tumorale à la graisse péri-vésicale chez 3 patients (Figure 3), soit 30% des cas, des métastases pulmonaires et ganglionnaires chez 3 malades, soit dans 30% des cas. La cystectomie totale associée à un curage ganglionnaire ilio-obturateur bilatéral a été réalisée chez 7 patients, suivie d´un geste de dérivation urinaire par Bricker chez 5 patients et une urétérostomie cutanée chez 2 patients dont l´état général était altéré, 4 malades ont bénéficié d´une chimiothérapie adjuvante, cependant la chimiothérapie palliative n´a été réalisée que chez 2 patients. Les suites opératoires immédiates étaient compliquées d´une collection intra-abdominale du côté de Bricker chez une patiente à J6 du postopératoire et d´un décès à J7 du post-opératoires par détresse respiratoire. L´évolution à long terme était favorable avec un recul de 3 ans chez 5 patients soit 50% des cas et défavorable chez 2 autres patients (rechute métastatique), décès chez un patient après refus de traitement palliatif. Deux patients ont été perdus de vue.

## Discussion

Le carcinome épidermoïde est une tumeur rare dans les pays occidentaux dont la fréquence et entre 2 et 5% et vient en deuxième position après le carcinome urothélial, cependant, cette tumeur est fréquente en pays du Moyen Orient, Sud-est d´Asie et en Amérique du sud dont la Bilharziose est endémique avec une fréquence de 20 à 30%. Il est le premier sous-type histologique en Egypte où il présente un problème de santé publique. Le mécanisme pouvant mener au développement d´un carcinome épidermoïde a été initialement proposé par MOSTOFI, qui a mis en évidence une réponse de l´épithélium vésical aux facteurs liés à l´environnement et irritatifs vésicaux comprenant 3 étapes: prolifération cellulaire, métaplasie, néoplasie, avec progression et mutation cellulaire progressives [3]. Plusieurs facteurs de risque sont établis; la Bilharziose urinaire, le tabagisme, les vessies neurologiques, la radiothérapie, les infections urinaires chroniques [5-7], la présence de corps étrangers intra-vésicaux et tous les obstacles qui sont susceptibles d´entraîner une stagnation urinaire chronique et qui correspondent à une irritation chronique de l´urothélium vésical. Une irritation vésicale chronique a été retrouvée chez 7 de nos 10 malades soit dans 70% des cas.

La présentation clinique du carcinome épidermoïde est dominée essentiellement par l´hématurie et les troubles mictionnels notamment la pollakiurie diurne ou nocturne, les brûlures mictionnels, on peut même trouver des douleurs pelviennes, des lombalgies en rapport avec l´extension tumorale [8]. En effet, les facteurs irritatifs vésicaux qui participent à la carcinogenèse tumorale sont eux-mêmes sources d´hématuries, de signes irritatifs et d´infections urinaires, masquant ainsi les éléments cliniques susceptibles de faire évoquer un processus tumoral débutant. A la cystoscopie, la lésion se présente généralement comme une tumeur compacte sous forme de nodule ou de plaque, avec un revêtement nacré dans les formes d´origine Bilharzienne et un aspect ulcérant dans les formes non Bilharziennes. Elle est le plus souvent unique, mais peut être diffuse dans certains cas.

Le diagnostic positif du carcinome épidermoïde repose principalement sur l´examen anatomo-pathologique d´une RTUV. La nature épidermoïde est affirmée par la présence d´une différenciation kératinisante et/ou l´existence de ponts intercellulaires. La coexistence d´un contingent à cellules transitionnelles fait porter le diagnostic de carcinome urothélial avec inflexion épidermoïde.

La cystectomie radicale associée à un curage ganglionnaire pelvien et une dérivation urinaire constitue le traitement de choix du carcinome épidermoïde assurant un contrôle de la maladie et une survie meilleure que la cystectomie partielle [8], la radiothérapie et la chimiothérapie. L´urètrectomie systématique est conseillée par Bejany *et al*. [4] qui a observé 40% de récidives urétrales après cystectomie. La radio-chimiothérapie concomitante peut être aussi comme alternative toujours utile en cas de tumeur non résécable ou dans le cas où la préservation de la vessie est désirée.

L´immunothérapie est une future approche qui semble présenter un complément prometteur du traitement chirurgical, vu son bénéfice qui apparaît indépendant du site de la tumeur primitive ou de son type histologique. Les principaux facteurs pronostiques du carcinome épidermoïde sont: le stade et le garde de la tumeur lors de la découverte. Le carcinome épidermoïde est caractérisé par une évolution à prédominance locorégionale; les métastases sont rares par rapport au carcinome urothélial, elles sont retrouvées dans 18% des cas et dans 8 à 10% des cas pour le carcinome épidermoïde Bilharzien et non Bilharzien successivement. Cependant, son pronostic n´est généralement pas favorable. La mise en évidence de cette tumeur à un stade avancé pourrait expliquer cela. En effet, la survie à 5 ans et estimée à 50-60% et à 33-48% pour le carcinome épidermoïde Bilharzien et non Bilharzien successivement [8].

## Conclusion

Le carcinome épidermoïde de la vessie est une tumeur rare, qui demeure méconnue dans les pays occidentaux. Le traitement de ces tumeurs repose sur la chirurgie, l´intervention de référence étant la cystectomie radicale avec lymphadénectomie. Le pronostic reste sombre, du fait d´un diagnostic tardif.

### Etat des connaissances sur le sujet

- Le carcinome épidermoïde de la vessie est une tumeur rare;
- Sa prise en charge thérapeutique est mal codifiée vu la rareté des cas rapportés dans la littérature.

### Contribution de notre étude à la connaissance

- Il faut penser au diagnostic de carcinome épidermoïde de la vessie même en absence de facteurs de risque connus de cette pathologie;
- Confirme la place de la cystectomie totale avec un curage ganglionnaire tenu comme traitement de référence de cette tumeur rare.
